# Changing Landscape in the Treatment of Adult Acute Lymphoblastic Leukemia (ALL)

**DOI:** 10.3390/cancers14174290

**Published:** 2022-09-01

**Authors:** Tina Künz, Alexander W. Hauswirth, Gabriele Hetzenauer, Jakob Rudzki, David Nachbaur, Normann Steiner

**Affiliations:** 1 Department of Internal Medicine V (Hematology and Medical Oncology), Medical University of Innsbruck, A-6020 Innsbruck, Austria; 2 Department of Medicine I, Division of Hematology and Hemostaseology, Medical University of Vienna, 1090 Vienna, Austria

**Keywords:** ALL, acute lymphoblastic leukemia, chemoimmunotherapy, antibody-based therapy, CAR-T cells, tyrosine-kinase inhibitors

## Abstract

**Simple Summary:**

The improved understanding of acute lymphoblastic leukemia has brought with it several new therapy strategies as well as novel treatment agents. The aim of our review was to create a well-arranged overview of the currently available treatment strategies for ALL.

**Abstract:**

Acute lymphoblastic leukemia (ALL) is a rare hematological malignancy characterized by proliferation and accumulation of premature lymphoid blasts. Depending on risk factors, the survival of acute lymphoblastic leukemia has significantly improved over the last decades. During the last years, measurable residual disease (MRD) assessment has evolved into one of the most sensitive markers for prognosis and risk of relapse. For this reason, measurable residual disease detection and monitoring count as standard evaluation in patients with acute lymphoblastic leukemia. Allogeneic stem cell transplantation is still the recommended treatment option for patients with high and highest risk profiles as well as for relapsed or refractory settings. The increased understanding of the pathomechanism and heterogeneity of acute lymphoblastic leukemia has led to the development of several novel therapeutic opportunities such as tyrosine-kinase inhibitors, antibody-based therapies and CAR-T cells with the aim of improving clinical outcomes. Furthermore, the major advances in disease understanding of ALL have led to the identification of different subgroups and better disease stratification. Even though novel therapy targets are constantly developed, acute lymphoblastic leukemia remains a challenging and life-threatening disease. To improve the historically unsatisfying result in therapy of adult acute lymphoblastic leukemia many clinical trials have recently been initiated to determine the optimum combination regimens of novel and old agents for adult acute lymphoblastic leukemia.

## 1. Introduction

Acute lymphoblastic leukemia is a rare and heterogeneous hematological malignancy characterized by the uncontrolled proliferation of premature lymphoid cells. In recent years, the development of treatment options has improved the outcome for adults with ALL. Historically, adult ALL has been known for poor prognosis, limited treatment options and low cure rates.

Response and long-time survival are determined by different prognostic factors, of which measurable residual disease is presumed to be the most important. Moreover, the gained understanding of genomics and pathophysiology has led to the implementation of subgroups and specialized therapies. Novel therapy opportunities such as tyrosine-kinase inhibitors targeting BCR::ABL1, CD19-directed chimeric antigen receptor (CAR) T-cell therapy, antibody–drug conjugates (inotuzumab-ozogamicin), monoclonal antibodies (rituximab) and bispecific antibodies (blinatumomab) have significantly improved the treatment options for adults with ALL. However, the outcomes of adult patients with ALL are still in need of improvement. The following review focuses on the current standards in ALL therapy and further possible directions.

## 2. Epidemiology, Clinical Presentation

ALL typically shows a bimodal distribution with the first peak at around the age of 5 years and the second in adulthood (around the age of 50). With an annual incidence of 1.1 per 100,000 in Europe, adult ALL is presumed to be rare [1].

In the beginning, the clinical presentation is typically non-specific, but most often acute. Most of the symptoms can be ascribed to hematopoietic insufficiency. Besides anemia, granulocytopenia, leukopenia and thrombocytopenia, splenomegaly and hepatomegaly are seen frequently [1,2].

In most instances, the cause of the disease is unknown, but there are some known risk factors. Besides some diseases characterized by DNA repair mechanism damage (e.g., ataxia-telangiectasia), patients with trisomy 21 are also more likely to develop ALL. Furthermore, radioactive radiation and exposure to myelotoxic chemicals, for instance, chloramphenicol or benzol, facilitate the development of ALL. Every now and then, ALL is seen as secondary neoplasia after radiotherapy and chemotherapy with special agents [1,2].

## 3. Diagnostic, Genetic and Prognostic Factors

Acute lymphoblastic leukemia is a genetically heterogeneous lymphoid neoplasm derived from T-cell or B-cell progenitors. Diagnosis and differentiation into subgroups are based on morphological, genetic and immunophenotypical characteristics [3].

Diagnostic evaluation of ALL consists of bone marrow aspiration and biopsy followed by a morphological analysis, immunophenotyping by flow cytometry, karyotyping and fluorescence in situ hybridization (FISH) to determine the BCR::ABL1 fusion gene or KMT2A gene rearrangements [1]. Genetic analysis is the most powerful tool for identifying genomic abnormalities for further risk determination and treatment choice. Furthermore, the SNP array belongs to standard routine diagnostics. FISH can identify the most frequent abnormalities, but is limited in the number of detectable alterations. Nevertheless, it is still the most commonly used technique in clinical practice. The implementation of next-generation sequencing (NGS) led to the determination of novel molecular entities [4,5].

The German Multicenter Study Group on Adult Acute Lymphoblastic leukemia (GMALL) differentiates between B-lineage ALL and T-lineage ALL, characterized by different immunophenotype and molecular genetics (Table 1). B-lineage ALL comprises approximately 75% of ALL cases, while T-lineage ALL accounts for about 25% of adult ALL cases. Multicolor flow cytometry (MFC) is used to differentiate B-cell from T-cell ALL and to determine cell surface markers for further antibody therapy. Furthermore, flow cytometry can be used for MRD detection. B-lineage ALL is typically defined by expression of CD19, CD20, CD22 and CD79a and T-cell ALL is known for CD3-positivity and a variable expression of CD1a, CD2, CD5, CD7, CD56 and TdT. B-cell ALL has many genetic subtypes characterized by chromosomal alterations and rearrangements. These chromosomal abnormalities can be used to identify different disease subtypes for risk stratification [1,2].

Moorman et al. evaluated the prognostic impact of chromosomal abnormalities in 625 patients with adult B-cell precursor ALL. The study revealed that patients with complex karyotypes and patients with low hypodiploidy/near triploidy show higher rates of relapse and death than do other patients (24% and 22%, respectively). Patients with BCR::ABL1 did not show an increased relapse rate or poorer OS, while patients with KMT2A-AFF1 had a higher relapse rate but not a poorer OS [6].

A trial conducted by Paietta et al. in 1229 patients showed improved risk assessment in adult BCR::ABL1-negative B-ALL due to molecular classification. The study demonstrated that cytogenetic anomalies worsen outcomes and that molecular subtypes show different responses to treatment [7].

The Philadelphia chromosome is the most common cytogenetic aberration observed in B-ALL with increased incidence with age [1].

The white blood cell count (WBC) at diagnosis is an important prognostic factor in patients with ALL. Cut-off values of 30 × 109/L for B-lineage ALL and 100 × 109/L for T-lineage ALL are mainly used for the prognostic valuation of WBC. The prognostic factors lead to the division into a standard-risk and a high-risk group. At least one adverse prognostic factor is necessary for the high-risk group [1,8].

## 4. Measurable Residual Disease (MRD) Monitoring

The application of diverse chemotherapeutics has significantly improved outcomes in adult patients with ALL. Although most ALL patients achieve CR after induction therapy, relapses are common. Persistent leukemic blasts with resistance to cytotoxic chemotherapy are the main cause of relapse [9,10].

Evaluation of MRD appears to be the most important prognostic tool in ALL. The development of highly sensitive MRD assays, for instance, real-time PCR or next-generation sequencing (NGS), may allow even better treatment decisions and better risk stratification. The primary purpose of MRD monitoring is to determine treatment response and risk for relapse [11].

It would seem that among those patients with detectable MRD, the level of MRD may also affect the prognostic implications. A study by Gökbuget et al. showed significantly poorer outcomes in patients with MRD-positive ALL, particularly at higher MRD levels [12].

MRD evaluation should be performed after the first cycle of induction, at the time of complete remission and subsequently every three to six months. Persistence or reappearance of MRD after induction is associated with a poorer prognosis. Furthermore, time to MRD-negativity is a clinically significant predictor of outcome [13].

## 5. First-Line Therapy of Acute Lymphoblastic Leukemia

**Figure 1:**
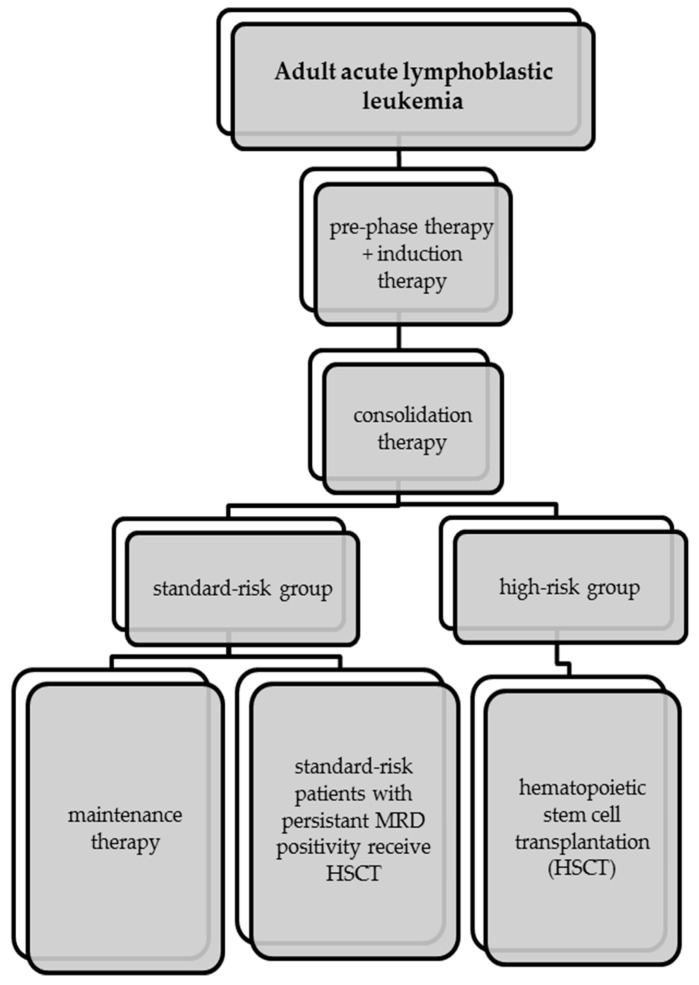
Standard treatment algorithm for adult Ph-ALL.

First-line therapy of Philadelphia chromosome-negative ALL is divided into three phases (Figure 1): induction, consolidation and maintenance. Before the actual start of first-line therapy, a pre-phase treatment with a total length of five days is administered to reduce the risk for tumor lysis syndrome [8]. Treatment of Philadelphia chromosome-positive ALL differs from first-line treatment of Philadelphia chromosome-negative ALL, since a tyrosine-kinase inhibitor is typically added to treatment [2,8].

The goal of induction therapy is to achieve complete remission. During the induction phase various drugs such as dexamethasone, vincristine, asparaginase and anthracyclines are used to eradicate disease and induce complete remission [2]. The induction phase is followed by the consolidation phase, where the elimination of residual leukemia cells and MRD reduction are the primary intentions [9]. After consolidation therapy the patients are divided into a high-risk and a standard-risk group, which is relevant for the following therapy steps [1,2].

Treatment is completed with maintenance therapy for up to 12–24 months. During maintenance therapy, the standard-risk patients receive weekly methotrexate and daily 6-mercaptopurine, while high-risk patients undergo hematopoietic stem cell transplantation [2,8].

The optimal treatment for adolescents and young adults with ALL has been intensely debated. However, there is clear evidence that demonstrates the superiority of pediatric regimens as compared to adult treatment regimens for adolescents and young adults [14,15,16].

## 6. CNS Prophylaxis

Because malignant B-cells as well as malignant T-cells tend to infiltrate cerebrospinal fluid (CSF) and the meningeal membranes in some patients already show central nervous system (CNS) involvement at presentation, a high WBC count at diagnosis, Philadelphia chromosome-positivity, T-cell immunophenotype, mature B-cell phenotype//Burkitt leukemia and the presence of a mediastinal mass are associated with CNS disease [17,18].

Consequently, all newly diagnosed patients with ALL should routinely undergo a lumbar puncture and first-line therapy should involve intrathecal CNS prophylaxis. Even though the incidence of CNS involvement at presentation is low (5–10%), the risk for CNS relapse is high if adequate CNS prophylaxis is not administered. Nowadays preventive CNS treatment has become a fundamental part of adult ALL treatment. Nevertheless, the optimal procedure for CNS therapy is currently unclear [17]. Multiple treatment modalities including systemic therapy, intrathecal therapy (IT), cranial radiation therapy and combinations thereof are used. Cranial irradiation is the oldest CNS prophylaxis procedure and is presumed to be very effective, but the rates of secondary neoplasms, neurotoxicities, neurocognitive dysfunctions and endocrinopathy argue against cranial irradiation [18,19].

Success in treating CNS disease with systemic chemotherapy depends on the ability of the cytotoxic drug to cross the blood–brain barrier. Due to insufficient therapeutic concentrations in CSF and potential toxicities, system chemotherapy alone is not an appropriate CNS prophylaxis. Adverse events after cranial radiation and limitations in achieving sufficient cytotoxic levels of systemic chemotherapeutics in the CSF lead to the implementation of IT chemotherapy in CNS prophylaxis protocols. Mainly used IT therapies are cytarabine (AraC) and methotrexate (MTX) [18].

At the present time, skull radiation belongs to standard therapy, but the GMALL study (08/2013) is currently evaluating the need for full-skull radiation in the standard treatment of ALL [19].

## 7. Antibody-Based Therapy

Although adults with ALL achieve high CR rates in the first-line setting, nearly half of them relapse [20]. Even with the most intensive therapy, the prognosis in the relapsed/refractory setting was historically poor with cure rates of less than 10% and a median overall survival of six months [12].

Thanks to the significantly improved outcome during studies, novel therapy opportunities targeting CD19, CD20, CD22 and CD52 surface antigens have become more important in ALL treatment over the past years (Table 2). Although most studies have focused on using these drugs in the refractory/relapse setting, their inclusion in first-line regimens could also be valuable. Some study groups are currently evaluating blinatumomab and inotuzumab ozogamicin for newly diagnosed primarily older patients with ALL [20].

### 7.1. Monotherapy with Blinatumomab in Relapsed/Refractory ALL

Blinatumomab, an anti-CD19/anti-CD3 bispecific T-cell-engaging (BiTE) antibody construct, is indicated for relapsed/refractory B-ALL. As seen from the high expression rates of CD19 on B-cell precursor ALL blasts, CD19 is a highly promising target for ALL therapy. Blinatumomab leads to lysis of CD19—expressing B-cells by recruiting CD3 cytotoxic T-cells. Blinatumomab has received FDA and EMA approval for the treatment of CD19-positive relapsed/refractory B-ALL [21].

In a randomized multi-institutional phase III trial (TOWER) blinatumomab was compared with standard chemotherapy for adults with Ph-negative relapsed/refractory B-cell ALL [22]. The primary endpoint was overall survival. A total of 405 patients were enrolled in the study, 271 of whom were grouped in the blinatumomab group and 134 in the standard chemotherapy group. The study showed significantly longer overall survival for the blinatumomab group. The median overall survival in the blinatumomab group was 7.7 months, while the standard-of-care chemotherapy group showed a median overall survival of 4.0 months. Furthermore, the blinatumomab group showed a significantly higher remission rate within the first 12 months after treatment initiation as compared to the chemotherapy group. Of the patients who achieved complete remission (full/partial or incomplete hematologic recovery), 48% in the standard chemotherapy group and 76% in the blinatumomab group achieved negative MRD status. The median duration of remission was 7.3 months for the blinatumomab group and 4.6 months for the chemotherapy group. The estimated six-month event-free survival was 31% for the blinatumomab and 12% for the chemotherapy group. Of the patients in the blinatumomab and also in the chemotherapy group, 24% underwent hematopoietic stem-cell transplantation. Adverse events were reported in 99% of the patients in the blinatumomab as well as the chemotherapy group. Of the patients in the blinatumomab group, 62% vs. 45% in the standard chemotherapy group developed serious adverse events such as neutropenia, infections, lymphopenia, cytokine-release syndrome (CRS), neurological events or elevated liver enzymes. Serious adverse events were reported in 19% of the patients in the blinatumomab group and in 17% of the patients in the chemotherapy group. This randomized trial shows the significant survival benefits of blinatumomab as compared with the standard-of-care chemotherapy for patients with Ph-negative relapsed/refractory ALL [22].

Blinatumomab for ALL relapse after allogeneic hematopoietic stem cell transplantation was tested in a phase II study [23]. A total of 189 patients with relapsed/refractory Philadelphia chromosome-negative B-ALL were enrolled, 64 of whom underwent previous allogeneic HSCT. Patients with previous allogeneic HSCT achieved a CR rate of 45% after blinatumomab therapy, while those without previous allogeneic HSCT had a CR rate of 43%. The MRD response rates were similar in patients with previous allogeneic HSCT and in patients who did not undergo allogeneic HSCT before blinatumomab therapy. In patients with previous allogeneic HSCT who showed CR during the first two cycles and MRD response, median relapse-free survival was 7.6 months. The median overall survival was 23.1 months. In patients with previous allogeneic HSCT who achieved CR but did not have a complete MRD response, the median relapse-free survival was 6.1 months and the median overall survival was 12.7 months. The results of this study demonstrate the effectiveness of blinatumomab for patients with relapsed/refractory Philadelphia chromosome-negative ALL after previous allogeneic HSCT [23].

### 7.2. Monotherapy with Inotuzumab Ozogamicin in Relapsed/Refractory ALL

Inotuzumab ozogamicin is a CD22 monoclonal antibody conjugated to calicheamicin. CD22 is a transmembrane glycoprotein and is expressed in more than 90% of patients with B-ALL. Currently, inotuzumab ozogamicin is indicated for the treatment of relapsed/refractory CD22-positive B-ALL [21].

A study by Bueno et al. demonstrated that CD22 precedes CD19 in normal B-cell development in early B progenitors. Furthermore, they demonstrated that preexisting CD34^+^CD19^−^CD22^+^ leukemic cells show a mechanism of resistance to CD19-targeted immunotherapies by phenotypic escape. They described a 1.7-fold higher frequency of CD34^+^CD19^−^CD22^+^ cells 30 days after CAR-T cell infusion, which demonstrated a relative increase in the proportion of CD34^+^CD19^−^CD22^+^ after the treatment-associated elimination of CD19+ blasts. Additionally, they evaluated the impact of CD34^+^CD19^−^CD22^+^ relapse risk. Patients with relapse after CD19-directed therapy showed a threefold higher expression of CD34^+^CD19^−^CD22^+^ cells. This gave rise to the assumption that CD34^+^CD19^−^CD22^+^ immunophenotyping at the time of initial diagnosis could be advisable [24]. During a randomized multicenter phase III study (INO-VATE), inotuzumab ozogamicin was compared with standard intensive chemotherapy for patients with relapsed or refractory ALL. A total of 218 patients were enrolled in the study and subsequently divided into 2 groups of 109 patients each. The defined primary endpoints were complete remission and overall survival [25]. The inotuzumab ozogamicin group achieved a significantly higher rate of complete remission or complete remission with incomplete hematologic recovery than did the chemotherapy group (80.7% vs. 29.4%, respectively). Among the patients with complete remission, the percentage of patients with MRD results below the threshold was significantly higher in the inotuzumab group. Moreover, the median duration of remission was 4.6 months in the inotuzumab ozogamicin group vs. 3.1 months in the standard group. The inotuzumab ozogamicin group showed significantly longer progression-free survival than did the chemotherapy group (5.0 months vs. 1.8 months, respectively). A significantly higher percentage of patients in the inotuzumab group underwent hematopoietic stem-cell transplantation directly after treatment. The most common hematologic adverse event in both treatment groups was cytopenia. The percentage of thrombocytopenia Grade 3 or higher was lower in the inotuzumab group (37%) than in the standard group (59%). Febrile neutropenia (Grade 3 or higher) was seen more often in the standard therapy group (49%) than in the inotuzumab group (24%). The percentage of patients with serious adverse events was similar in both treatment groups. Inotuzumab was associated with more hepatotoxicity and veno-occlusive disease (VOD), but hematologic and infectious complications were less frequent [25].

### 7.3. Chemoimmunotherapy

Due to the fact that novel antibody constructs showed promising results and efficacy during single-agent studies, several study groups are currently investigating the combination of antibodies with standard chemotherapy. Table 3 shows the published combination studies for adult Ph-negative ALL.

### 7.4. Chemoimmunotherapy with Inotuzumab Ozogamicin in Relapsed/Refractory ALL

The combination of low-intensity chemotherapy (mini-hyper-CVD) plus inotuzumab ozogamicin with or without blinatumomab was investigated in a study with relapsed/refractory Philadelphia chromosome-negative ALL patients. The results were encouraging with an MRD-negativity rate of 82% and a CR/CRi rate of 78%. Treatment cycles with odd numbers included cyclophosphamide (days 1–3; 150 mg/m^2^ every 12 h), dexamethasone (days 1–4 and 11–14; 20 mg/d) and vincristine (days 1 and 8; 2 mg flat dose). Even numbered cycles comprised of methotrexate (day 1; 250 mg/m^2^) and cytarabine (days 2 and 3; 0.5 g/m^2^ every 12 h). The overall response rate was 80%. Median overall survival was 25 months in salvage chemotherapy 1, six months in salvage chemotherapy 2 and seven months or more in salvage chemotherapy 3. The historical comparison showed a significant improvement in median overall survival as compared to inotuzumab ozogamicin as a single agent (14.0 months vs. 6.0 months). The study showed a VOD incidence of 15% [26].

Fifty patients with relapsed/refractory CD22+ ALL were enrolled in a phase I trial of inotuzumab in combination with cyclophosphamide, vincristine and prednisone (CVP) [18]. The CR/CRi rate was 61%. Median overall survival was 7.7 months for all patients and 10.9 months for the patients treated with the maximum tolerated dose (MTD) [27].

### 7.5. Chemoimmunotherapy with Blinatumomab in Newly Diagnosed B-Cell ALL

Another phase II trial verified the combination of blinatumomab with hyper-CVAD (cyclophosphamide, vincristine, doxorubicin and dexamethasone) for patients with newly diagnosed B-cell ALL [28]. The treatment protocol includes four cycles of hyper-CVAD, high-dose methotrexate/cytarabine and four following cycles of blinatumomab. Twenty-seven patients were enrolled in this trial. The reported overall complete remission rate was 100%. Of the treated patients, 96% achieved MRD-negativity. During the median follow-up at 17 months, two deaths and four patients with relapse were reported. The estimated 12-month relapse-free survival rate was 76% and estimated overall survival was 89%. There was one reported Grade 3 CRS and four patients developed a Grade 3–4 neurological adverse event related to blinatumomab. Based on its safety and high effectiveness, the combination of hyper-CVAD and blinatumomab appears to be promising [28].

### 7.6. Chemoimmunotherapy with Rituximab

Rituximab is an anti-CD20 antibody used for the treatment of CD20+ adult B-cell ALL. CD20 is a B lymphocyte-specific integral membrane phosphoprotein, expressed on normal and malignant B-lymphocytes, but not on normal stem cells [21].

Standard chemotherapy with or without rituximab for patients with Ph-negative CD20+ ALL was tested in the GRAALL trial. In total, 209 patients were enrolled, 105 patients were assigned to the rituximab group and 104 to the control group. Due to the lower incidence of relapse in the rituximab group, the rituximab group showed longer event-free survival than the control group. The superiority of rituximab regarding event-free survival did not lead to significantly longer overall survival. The rate of complete remission after induction was 92% in the rituximab group and 90% in the control group. Of the 209 patients, 124 showed adverse events, with no significant difference between the two groups [29].

From 1992 to 2009, a study investigated the combination of rituximab with standard/modified hyper-CVAD for patients with de novo Philadelphia chromosome-negative B-ALL [30]. Standard hyper-CVAD included fractionated cyclophosphamide, vincristine, doxorubicin and dexamethasone. There were two different modified hyper-CVAD modalities. Hyper-CVAD 1 included anthracycline intensification with or without rituximab, while hyper-CVAD 2 abstained from anthracycline. The overall CR rate was 95%, with lower CR rates in the older group. MRD-negativity was seen more often in the CD20-positive subgroup treated with rituximab than in the CD20-negative group. With a median follow-up of 64 months, the overall 3-year CR duration was 60% and the overall survival rate was 58%. Younger age, low WBC and rituximab therapy were identified as predictors of a favorable outcome. Treatment with modified hyper-CVAD 2 showed improved 3-year CR duration as compared to the other groups (78% vs. 53% and 54%, respectively). The 3-year overall survival was 60% for the hyper-CVAD 2 group and 55% for the hyper-CVAD 1 and the standard hyper-CVAD group. There was no increase in induction mortality for the CD20-positive subgroup [30].

Chemoimmunotherapy with hyper-CVAD and rituximab for adult Burkitt and Burkitt-type lymphoma or ALL were investigated in a study from 2000 to 2005 [31]. Thirty-one patients either untreated or following one cycle of chemotherapy were included. The treatment included eight alternating chemotherapy courses every 21 days. Rituximab was administered during courses 1 to 4. Of the patients, 86% achieved CR. There were no reported induction deaths. With a median follow-up of 22 months, 7% (2/28 patients) relapsed; 3-year overall survival was 89%. The results of this trial were compared with the historical results for hyper-CVAD alone. The comparison showed similar CR rates but improved 3-year estimated survival, event-free survival, disease-free survival and reduction in induction mortality for patients treated with hyper-CVAD plus rituximab [31].

### 7.7. Chemoimmunotherapy with Ofatumumab

Ofatumumab, an anti-CD20 antibody binds to a novel epitope of CD20. During a phase II study, ofatumumab was evaluated in the treatment of patients with newly diagnosed untreated/minimally treated Ph-negative CD20-positive B-ALL. Between 2011 and 2017, 69 patients were enrolled in the study. The primary endpoint of the study was relapse-free survival and secondary endpoints were CR rate, MRD-negativity rates and overall survival. Of the patients, 98% achieved CR and the MRD-negativity rate was 65% after Cycle 1. The most common non-hematologic toxicities of Grades 3–4 were infections. Overall survival and median relapse-free survival were 52 months and the estimated 4-year relapse-free survival and overall survival were 60% [32].

A phase II trial compared hyper-CVAD plus ofatumumab with hyper-CVAD plus rituximab in patients with newly diagnosed Philadelphia chromosome-negative B-ALL [33]. The treatment was based on a hyper-CVAD regimen alternating with high-dose methotrexate and cytarabine every three weeks. Rituximab and ofatumumab were given for a total of eight doses. A total of 222 patients were enrolled, 69 patients were divided into the hyper-CVAD plus ofatumumab group, 95 patients into the hyper-CVAD plus rituximab group and 58 into the hyper-CVAD group. Additionally, the study differentiated between patients with a CD20 expression ≥20% and ≤20%. The overall CR rate after induction therapy was 95%. The CR rate in the hyper-CVAD plus the ofatumumab group was 93%, while the CR rate in the hyper-CVAD plus the rituximab group and the hyper-CVAD group was 97%. After induction therapy, 63% of the patients achieved MRD-negativity. In those patients with a CD20 expression ≥20%, the 4-year EFS rate was 61% in the ofatumumab group and 43% in the rituximab group. Patients with hyper-CVAD plus ofatumumab achieved a 4-year OS of 67% and patients with hyper-CVAD plus rituximab 51%. The 4-year EFS among the patients with CD20 expression ≤20% was 65% with hyper-CVAD plus ofatumumab and 50% with hyper-CVAD plus rituximab. The 4-year OS rates were 70% with ofatumumab and 62% with rituximab. In summary, hyper-CVAD plus ofatumumab was associated with better outcomes, especially in younger patients with newly diagnosed ALL. Furthermore, ofatumumab was well-tolerated [33].

### 7.8. Chemoimmunotherapy with Epratuzumab

Epratuzumab is a humanized CD22 antibody that binds to the extracellular domain of CD22. CD22 is expressed on more than 90% of ALL blasts. In a phase II trial, epratuzumab in combination with clofarabine and cytarabine was tested for patients with relapsed/refractory ALL. A total of 31 patients were evaluable for the study; ten achieved CR and six CRi. Only six patients with CR/CRi underwent MRD assessment at both time points and one of these patients showed MRD-negativity. The study showed a median overall survival of five months with a median follow-up time of four months. The response rate of 52% (CR/CRi) during this study is encouraging and prompts the assumption that epratuzumab is a promising agent in ALL treatment [34].

## 8. Therapy Opportunities for Older Adults

Adults older than 60 years of age with ALL show higher relapse rates, heightened early mortality and poorer prognosis in general. The challenge of ALL therapy in older adults is to ensure maximum therapy efficacy while simultaneously reducing early treatment-related mortality. Consequently, the currently valid recommendation is dose-reduced chemotherapy, antibody-based therapy or molecular therapy [35]. Table 4 demonstrates the currently published combination trials for patients over the age of 60.

A currently ongoing trial (EWALL-INO) (NCT03249870) is evaluating the combination of inotuzumab ozogamicin plus mini-hyper-CVD for older patients (older than 55) with newly diagnosed ALL. So far, the combination of inotuzumab ozogamicin with mini-hyper-CVD seems to be a safe and active therapy option for first-line therapy of older adults [36].

**Table 4:** Combination trials for first-line Ph-negative ALL (older adults).

Regimen	Study Population	N	Median Age	CR/CRi Rate	CR Duration	**MRD-** **Negativity**	**OS Rate**	Reference
Inotuzumab + mini-HCVD ± blinatumomab	Patients aged ≥60 years	64	68	98%	76% (3-year)	95%	54% (3-year)	[37]
Blinatumomab + POMP	Patients aged >60 years	31	73	66%	DFS 56% (1-year)	92%	65% (1-year)	[38]

HCVD hyper-fractionated cyclophosphamide, vincristine, dexamethasone; N number; CR complete remission; CRi complete remission with incomplete hematologic recovery; MRD measurable residual disease; OS overall survival; DFS disease-free survival; POMP prednisone, vincristine, methotrexate, mercaptopurine.

A phase II study investigated the combination of inotuzumab ozogamicin plus mini-HCVD with or without blinatumomab in the first-line setting for older adults with newly diagnosed Philadelphia chromosome-negative B-Cell ALL [37]. A total of 73 untreated patients were enrolled in the study. The patients received mini-hyper-CVD consisting of cyclophosphamide, vincristine, dexamethasone (50% dose-reduced), methotrexate (75% dose reduction) and cytarabine for up to eight cycles. Of the 73 patients, 70 were evaluable for efficacy, while 64 of the patients were evaluable for morphologic response with 63 of them showing a response. Additionally, 53/66 patients achieved MRD-negativity after the first cycle and, in total, 65/68 patients achieved MRD-negativity. Of the patients who achieved remission, 13% relapsed, 4% underwent allogeneic SCT in first remission and 32% died in CR. The 4-year overall survival rate was 61% for patients aged 60–69 vs. 34% in patients older than 70 years. The rate of death in remission was higher in older patients (45% vs. 20%, respectively). The remission deaths of the older patients were primarily associated with infections or the development of MDS/AML. With an overall response rate of 98% and a 4-year overall survival rate of 50%, the combination of hyper-CVD plus inotuzumab ozogamicin with or without blinatumomab appears to be safe and effective in the first-line treatment setting of older adults with newly diagnosed ALL [37].

A phase II trial evaluated blinatumomab followed by POMP (prednisone, vincristine, methotrexate, and 6-mercaptopurine) for older patients with newly diagnosed Philadelphia chromosome-negative B-cell ALL [38]. Thirty-one patients were enrolled and 29 were eligible for the trial. The achieved overall response rate was 66%. The estimated overall survival rate at six months was 68% and at 1 year, 56%. The most common non-hematologic toxicities of Grades 3–5 were hyperglycemia (14%), dyspnea (10%), hypertension (10%), febrile neutropenia (10%) and lung infections (7%). Overall, blinatumomab was effective in the first-line treatment of newly diagnosed elderly patients and was well tolerated [38].

## 9. Therapy of T-ALL

T-Cell ALL is an aggressive subtype of ALL. T-Cell ALL is less common than B-cell ALL. T-ALL can be divided into subgroups by intrathymic differentiation including pro-T, pre-T, cortical T, early T progenitor and medullary T-ALL. Each subgroup shows a variable expression of CD1a, CD2, CD3, CD4, CD5, CD7 and CD8 [39,40].

Prognostic factors are less clear in T-ALL patients than in B-ALL patients. Several risk factors used in B-ALL, including WBC and age, do not have the same prognostic value in T-ALL. As well as in patients with B-ALL, the MRD response is known to be the key prognostic factor [39].

The genetics of T-ALL is highly heterogeneous with chromosomal abnormalities in nearly all patients [39]. A large number of identified genes, including NOTCH1, PHF6, FBXW7, USP7, PTEN and others, act as oncogenic drivers in T-ALL [39]. Besides the mentioned mutations, T-ALL frequently shows chromosomal translocations of T-cell receptors, leading to abnormal expression of transcription factor oncogenes (LMO1, LMO2, TAL1, TLX1 and TLX3). Furthermore, the genetic diversity of T-ALL also includes chromosomal rearrangements (CALM-AF10, MiLL1-ENL, and NUP214-AFBL1), loss of transcription factors (e.g., LEF1, WT1, RUNX1, ETV6, etc.), cell cycle inhibitors (e.g., CDKN2A, RB and CDKN1B) and oncogene gains (e.g., MYB) [39].

NOTCH receptors act as oncogenic drivers in a multiplicity of cancer diseases, most notably in T-ALL. The NOTCH1 pathway regulates T-cell development and proliferation. NOTCH1 mutations are found in 50–60% of patients with T-ALL. Although available NOTCH-blocking drugs showed promising anti-tumor activity in preclinical studies, they are not effective in every patient and often cause side effects. In mouse models, NOTCH1 mutations did not initiate T-ALL, but their collaboration with oncogenes such as TAL1/SCL, ZMIZ1 and LMO2 led to the induction of leukemia. A preclinical study by Rakowski et al. demonstrated the interaction of ZMIZ1 with NOTCH1 and its potential as an oncogene. Therefore, ZMIZ1 may be a clinically relevant therapeutic target in T-ALL treatment [41]. Luo et al. evaluated Foxp3 and NOTCH1 in vivo as well as in vitro. In the study, NOTCH1 and Foxp3 expressions were higher in T-ALL mice than in normal mice [42].

BCL-2 proteins act as key regulators in intrinsic apoptosis. A study by Alford et al. demonstrated high expression levels of BCL-2 by primary ALL cells. The ALL cell cultures used in the study showed high sensitivity to BCL-2 inhibitors ABT-263 and ABT-199. Furthermore, the inhibition of BCL-2 led to rapid apoptotic death of ALL cells [43]. The first developed drugs targeting the BCL-2 pathway are venetoclax (BCL-2 inhibitor) and navitoclax (BCL-2/BCL-x inhibitor). Further clinical research is needed to determine the value of BCL-2 inhibition in T-ALL. Venetoclax in combination with navitoclax and chemotherapy was evaluated in a single-arm phase I study with 53 relapsed/refractory ALL patients. The overall CR rate was 59.6% in adult patients. The response rates were promising and median OS showed results comparable with other ALL targets (median OS for T-ALL 6.6 months). Overall, the study regimen was well tolerated. Study results showed the potential of venetoclax in combination with low-dose navitoclax and standard chemotherapy for patients with B- and T-ALL [44].

Nelarabine belongs to the deoxyguanosine analogue that selectively accumulates in T-cells, which makes it an auspicious agent in the management of T-ALL [39,40]. Nelarabine as a single agent was examined in two phase 2 trials. The trials showed CR rates of 31–36% and 1-year overall survival rates from 24 to 28% [45,46]. Nelarabine in combination with Hyper-CVAD was recently evaluated in a frontline setting for T-ALL: In 55 patients, the overall response rate was 96%. Of the patients, 31% relapsed with a median time to relapse of 7.3 months [47].

## 10. Therapy of Philadelphia Chromosome-Positive ALL

The Philadelphia chromosome (Ph) is the most commonly documented chromosomal abnormality in adult ALL. The incidence of Ph+ ALL increases with age, reaching a quotient of 50% in ALL patients older than 60 years [48]. Historically, the outcome for patients with Ph+ ALL was poor with a long-term survival rate of less than 20%. Besides CR, complete molecular response (CMR) is one of the main therapy goals in the treatment of Ph+ ALL. Formerly, Ph+ ALL patients were medicated with standard chemotherapy regimens, which led to high rates of complete remission after induction, but remission was short and the prognosis was still poor. The improved understanding of the pathophysiology of Ph+ ALL enabled the development of the first tyrosine kinase inhibitors (TKI). Multiplicities of studies have explored the combination of TKIs with intensive/dose-reduced chemotherapy, steroids or antibodies (Table 5).

Imatinib was the first tyrosine kinase inhibitor used in the treatment of patients with Ph+ ALL. Its implementation in chemotherapy regimens led to improved CR rates and long-time overall survival [2,48].

A phase II study evaluated imatinib in combination with hyper-CVAD for the first-line setting of adult patients with Ph-positive ALL [49]. Patients with newly diagnosed ALL (*n* = 54) were enrolled and treated with hyper-CVAD plus imatinib; 93% of the patients achieved CR. Of the patients who achieved CR, 95% achieved it after one course of therapy and 5% after a second course. At a median of 4.1 weeks, 88% of the patients achieved MRD-negativity. Median overall survival was 31 months with an estimated 2-year overall survival rate of 57% and an estimated 5-year overall survival rate of 43%. Median disease-free survival for patients in CR was 22.0 months [49].

Between 1993 and 2003, 266 patients were enrolled in the UKALLXII/ECOG2993 study for adults with Philadelphia chromosome-positive ALL [50]. The study evaluated the addition of imatinib to standard treatment regimens for newly diagnosed Ph-positive ALL. The overall CR rate was 92%. At four years, the overall survival rate of the imatinib patients was 38%, the relapse-free survival rate 50% and the event-free-survival rate 33%. This gives rise to the assumption that the addition of imatinib leads to significantly better outcomes than without. Furthermore, the study attempted to determine whether the outcome is dependent on application time. The study showed that there is no significant difference in outcome between the early and the late imatinib cohorts [50].

A further randomized study compared 268 adults with Ph-positive ALL in two arms: Arm A consisted of high-dose imatinib and reduced-intensity chemotherapy vs. Arm B with standard imatinib dose and hyper-CVAD [51]. The CR rate in Arm A was higher than in Arm B (98.5% vs. 91.0%). After the second cycle, 65.4% of the patients achieved a major molecular response (MMolR). Patients in Arm A showed a longer EFS (2.5 vs. 1.8 years) and longer OS (4.1 vs. 3.3 years) than did patients in Arm B. Compared to the pre-imatinib era, a higher percentage of the patients were brought to stem cell transplantation (63% allogeneic, 14% autologous) [51].

Dasatinib is a second-generation TKI that was combined with intensive chemotherapy in a phase II study (Table 6). The study showed a CR rate of 96%, a 5-year relapse-free survival rate of 44% and an overall survival rate of 46%. Moreover, 94% of the study cohort showed MRD-negativity at a median of three weeks [52]. Furthermore, in combination with intensity-reduced chemotherapy, Dasatinib was examined in two studies and showed CR rates of 96–100% (Table 6). The 5-year relapse-free survival rate was 37% and 28% and the 5-year overall survival rate was 46% and 36% in the two studies [53].

Ponatinib is a third-generation BCR::ABL1 inhibitor with activity against T315I mutations, a common mechanism of resistance to previous-generation TKIs. The long-term safety and efficacy of ponatinib and hyper-CVAD as first-line therapy for patients with Ph+ ALL have been evaluated in a study [55] that enrolled and treated 86 patients. The 3-month complete molecular response rate (CMR) was 74% and the overall CMR rate was 84%. At a median follow-up of 43 months, 71% of the patients are still alive and in remission. The 3-year event-free survival rate was 70% and the overall survival rate was 78%. The 5-year event-free survival rate was 68% and the 5-year overall survival rate was 73%. After a median of 20 months, 11 patients (13%) had relapsed. In general, the treatment was well tolerated and the most commonly seen adverse events were Grade 1 or 2. The combination of ponatinib and hyper-CVAD resulted in a durable response [55].

TKI implementation led to significantly improved outcomes for patients with Ph+ ALL; although, the outcome of relapsed patients is still poor. Based on this finding antibody-targeted therapies for the treatment of relapsed/refractory Ph+ ALL were analyzed in different studies.

For the treatment of patients with relapsed/refractory Ph+ ALL, blinatumomab was examined in a multicenter phase II trial that enrolled 45 patients. The achieved CR rate was 36%, with 88% of responders achieving MRD-negativity. The median overall survival was 7.1 months [56].

The safety and efficacy of blinatumomab in combination with a TKI were tested in a study with a small study population of 15. The study showed CR rates of 50% and 75% molecular response. The 6-month and 1-year overall survival rates were 73%. There were two reported cases of Grade 2 cytokine release syndrome. No cardiovascular adverse event was observed [57].

A multicenter phase II study examined the combination of dasatinib and blinatumomab for the first-line treatment of adult Ph+ ALL [58]. Between 2017 and 2019 63 patients with p190 or/and p210 were enrolled in the first chemo-free induction-consolidation protocol study for Ph+ ALL. The primary endpoint of the trial was the molecular response and secondary end-points were a reduced level of MRD, disease-free survival, overall survival and cumulative incidence of relapse. The CR rate was 98%. There was no significant difference in molecular response between the patients with p190 rearrangement and those with p210 rearrangement. The 12-month overall survival rate was 94.2% and the disease-free survival rate was 87.8%. Sixty adverse events occurred in 28 patients during therapy. The adverse events of Grade 3 or higher were cytomegalovirus (CMV) infection or reactivation, neutropenia, persistent fever, neurologic disorder and pulmonary hypertension. The response and survival rates for the combination of blinatumomab and dasatinib appeared to be highly promising for patients with Ph+ ALL [58].

A Phase I/II study evaluated the safety and efficacy of the combination of ponatinib, venetoclax and dexamethasone for patients with relapsed/refractory Philadelphia chromosome-positive ALL [59]. The tyrosine-kinase inhibitor ponatinib is highly active in Ph+ ALL, but, historically, single-agent responses are restricted and short-lived. So far, eight patients have been enrolled and six patients have already been treated. Three patients received 400 mg venetoclax daily and three patients received 800 mg venetoclax. The maximum tolerated dose has not yet been reached and no dose-limiting toxicities have been observed. Of the patients, 50% achieved complete remission. At a median follow-up of 8.6 months, median overall survival has not been reached and the estimated 9-month overall survival rate is 60% [59].

## 11. Chimeric Antigen Receptor (CAR) T Cell Therapy

Chimeric antigen receptor (CAR) T cell therapy targeting CD19 is a novel immunotherapy option for relapsed/refractory B-cell ALL, and has already shown promising clinical results and high efficacy. The mechanism involves genetically modified autologous cells targeting CD19-positive hematologic malignancies. Before every CAR-T cell infusion patients have to undergo lympho-depleting chemotherapy [60].

A phase II study evaluated the efficacy of a single infusion of tisagenlecleucel, a CD19-directed CAR-T cell therapy comprising two co-stimulatory domains, 4-1BB (CD137) linked to CD3 zeta [61]. The study enrolled 75 children and young adults aged 25 years (AYA—Adolescent and Young Adult) with relapsed or refractory CD19+ B-ALL. The primary endpoint was overall remission within three months. The overall remission rate achieved within three months was 81%, and all patients with response showed MRD-negativity. The 6-month event-free survival (EFS) and overall survival rates were 73% and 90%, and the 12-month EFS and OS rates were 50% and 76%, respectively. During this trial, tisagenlecleucel detection reached 20 months. Of the patients, 73% developed treatment-related Grades 3–4 adverse events, and 77% developed a cytokine-release syndrome (CRS). Neurological events occurred in about 40% of the study patients. This study of single-infusion of tisagenlecleucel showed durable remission and long-term persistence in pediatric and AYA patients [61].

Fifty-three adults with relapsed B-ALL were enrolled in a phase I trial and treated with an infusion of autologous T-cells expressing the 19-28z CAR, consisting of a different co-stimulatory domain than 4-1BB [62]. In this case, the second co-stimulatory domain was formed by CD28. The complete remission rate was 83%. At a median follow-up of 29 months, median EFS was 6.1 months and median overall survival was 12.9 months. The most common treatment-related non-hematologic adverse event was cytokine-release syndrome; 26% of the patients developed CRS. At the time of T-cell infusion, 32 patients suffered from a high disease burden. High tumor burden correlates with a higher incidence of CRS and, therefore, with neurologic events [63,64]. The adverse events coincide with shorter long-term survival, entirely contrary for patients with a low disease burden. During this study, pretreatment disease burden turned out to be a valuable predictor of survival and remission duration. The study showed favorable long-term remission rates for adult patients with a low disease burden after treatment with 19-28z CAR-T cells [62].

Adults with ALL face a significantly poorer prognosis than do children or AYAs, resulting in a relapse rate of 50% on currently available therapies. In October 2021, the FDA approved Tecartus as the first and only CAR T therapy for adults with relapsed or refractory B-cell ALL. It is based on the results of the ZUMA-3 trial, a global multicenter, single-arm, open-label study in which 65% of evaluable patients (N = 54) achieved CR at a median follow-up time of 12.3 months. Adverse events were seen up to Grade 3 or higher, such as CRS and neurologic events (ICANS—immune effector cell-associated neurotoxicity syndromes) in 26% and 35%, respectively. Hence, the Tecartus U.S. Prescribing Information received a BOXED Warning for the risk of CRS and neurologic syndromes and is consequently approved with a Risk Evaluation and Mitigation Strategy (REMS). Phase 1 data demonstrated that 32% of responders of a quite small number of enrolled patients (*n* = 22) are still in remission after a median follow-up time of 22.1 months [65]. The phase 2 data presented at the ASCO 2021 reported that 31% of the patients were in ongoing response at a median follow-up time of 16.4 months. The overwhelming aspect of these patients is that 97% of responders had a deep molecular response (MRD-negativity), and the median OS among all responders has not yet been reached [66].

## 12. Hematopoietic Stem Cell Transplantation

Hematopoietic stem cell transplantation (HSCT) is a well-established procedure in which patients with depleted bone marrow receive healthy hematopoietic stem cells. Historically, an allogeneic hematopoietic stem cell transplant was recommended in standard consolidation therapy for all eligible patients with ALL. Allogeneic HSCT reduces the risk of relapse and improves the survival rates of high-risk patients. Besides allogeneic HSCT, autologous hematopoietic stem cell transplantation is an available option, but several studies have shown the superiority of allogeneic HSCT in leukemia-free survival compared to autologous HSCT [67,68].

Allogeneic HSCT is typically indicated for patients with poor/high risk. Poor risk is generally defined as the presence of a high white blood cell count, unfavorable cytogenetic abnormalities, older age and late response to induction therapy [68].

A multicenter randomized study of adults with high-risk ALL compared three different post-remission therapy options [69]: chemotherapy, allogeneic HSCT and autologous HSCT. A total of 222 high-risk ALL patients were enrolled and 182 of them achieved complete remission. Of the 182 patients with CR, 84 were grouped in the matched sibling donor (MSD) group, 48 in the chemotherapy group and 50 in the autologous HSCT group. Disease-free survival and overall survival did not significantly differ between the different groups. The 5-year disease-free survival rate was 35% and the overall survival rate was 34%. This study did not show any superiority of family donor allogeneic HSCT over autologous HSCT or chemotherapy in high-risk ALL patients [69].

Goldstone et al. evaluated the role of allogeneic stem cell transplantation for adults with ALL as compared to autologous stem cell transplantation with standard chemotherapy in a multicenter randomized study that enrolled 1929 patients [70]. Patients with available MSDs (matched sibling donors) were grouped in the allogeneic HSCT group, while patients without MSD were assigned to the chemotherapy and autologous HSCT group. At 5 years, patients with MSD showed significantly improved overall survival (53% vs. 45%) and even a lower relapse rate, as compared to the patients who underwent chemotherapy and autologous HSCT. Patients at high risk did not show a significant difference in overall survival (41 vs. 35%), but the difference in patients with standard risk was significant. Furthermore, the relapse rate was significantly reduced in both risk groups after allogeneic transplantation [70].

A further study evaluated the outcome of patients with ALL in first complete remission with a sibling donor or no donor; 288 patients were included [71]. In the donor group, 95% (91 of 96 patients) of the patients received an allogeneic HSCT from an HLA-identical sibling donor, three patients received an autologous HSCT, while the remaining two did not undergo HSCT. In the no donor group, 123 of 161 (76%) of the patients underwent autologous HSCT, 31 (19%) patients received allogeneic HSCT from a mismatched related (*n* = 2) or unrelated donor (*n* = 29) and seven (4%) patients did not receive HSCT. The donor group showed a significantly reduced risk for relapse in comparison to the no donor group. The reduced risk for relapse was especially seen in the standard-risk group, and even in the high-risk group. The reduction was significantly higher in the donor group. The donor group showed improved overall survival (61% vs. 47%) at five and eight years as compared to the no donor group. This study showed a favorable effect of allogeneic HSCT, especially for patients with a standard-risk profile [71]. In conclusion, allogeneic HSCT still plays a fundamental role in ALL treatment, especially in high-risk ALL patients.

A retrospective single-center analysis attempted to identify whether or not early consolidation with autologous stem cell transplantation is feasible without reduced efficacy [72]. For this purpose, 59 patients with newly diagnosed B- or T-precursor ALL were enrolled and underwent either autologous or allogeneic HSCT. Twenty-four standard-risk patients received autologous HSCT and thirty-five high- and highest-risk and relapsed/refractory patients underwent allogeneic HSCT. The median time from diagnosis to HSCT was 4.5 months. Of the patients, 51% received a graft from an HLA-identical sibling donor, while 34% received their transplant from an HLA-matched unrelated donor and 14% from an HLA-mismatched unrelated donor. The primary endpoint was overall survival. Secondary endpoints were disease-free survival, relapse incidence and non-relapse mortality. The 10-year overall survival and disease-free survival rates for standard-risk patients in the autologous HSCT group in first complete remission were 45% and 33%, respectively. Thirteen patients relapsed, with two of them showing a late relapse more than two years after autologous HSCT. The cumulative relapse incidence in this group was 56% at 5 years and 67% at 8 years. The non-relapse mortality after autologous HSCT was 0%. High- and highest-risk patients in first complete remission or a relapsed/refractory setting who underwent allogeneic HSCT showed a 10-year overall survival rate of 58% and a 10-year disease-free survival rate of 55%. The 2- and 5-year non-relapse mortality rate was 12%. The cumulative relapse incidence was 30%. Acute graft-versus-host disease (GvHD) of Grade 2 or higher occurred in 34% of the patients. The presence of acute GvHD was associated with a significantly improved 5- and 10-year overall survival (92% vs. 42%) due to a lower relapse incidence (13% vs. 38%). Of the cohort, 22% developed chronic graft-versus-host disease. Patients with chronic GvHD tended to have better overall survival (71% vs. 55%) and better disease-free survival (71% vs. 50%) [72].

On the basis of evidence, patients with a standard-risk ALL and MRD level below 0.01% at end-of-induction or end-of-consolidation are treated with conventional chemotherapy, while those with poor MRD results undergo HSCT. Ribera et al. evaluated whether therapy decisions for adult Ph-ALL patients with poor-risk features should be based on the MRD level. In their study, they demonstrated that patients with high-risk features and early MRD response definitely benefit from chemotherapy without HSCT [73].

## 13. Conclusions

The treatment outcome of patients with ALL has significantly improved mainly due to optimized and individualized therapy strategies and the optimization of supportive care. The accurate definition of subgroups based on prognostic factors, cytogenetic and molecular markers has enabled the implementation of risk-adapted and individual therapy steps. Furthermore, the development of novel therapies targeting leukemic cell surface antigens has been a milestone in the treatment and management of ALL. Future investigations will show the role of immunotherapies in ALL therapy, especially in the frontline setting. Additionally, they will determine whether the use of antibody-based therapy options in frontline settings will significantly improve clinical outcomes.

Allogeneic hematopoietic stem cell transplantation remains the gold standard for relapsed/refractory patients without MRD-negativity and Ph+ ALL patients.

## Figures and Tables

**Table 1:** Subgroups in adult ALL.

Subgroup	Immunophenotype	Cytogenetic, Molecular Genetics
**B-Lineage ALL**	HLA-DR^+^, TdT^+/−^, CD19^+^, cyCD79a^+^, cyCD22^+^	
Pro-B-ALL	CD10^-^	t(v;11), KMT2A rearrangements
c-common-ALL	CD10^+^	t(9;22), BCR::ABL1; IKZF1
Premature B-ALL	cyIgM^+^	t(1;19)TCF3::PBX1; t(9;22); BCR::ABL1 IKZF1
Mature B-ALL	TdT^−^, CD34^−^, sIg^+^	t(8;14); MYC rearrangements
**T-lineage ALL**	TdT^+/−^, cyCD3^+^, CD7^+^	
Pro-T-ALL	cCD3^+^, sCD3^−^, CD1a^−^, CD2^+^, CD5^−^, CD7^+^, CD34^−^	NOTCH1/FBXW7 mutations
Pre-T/immature T-ALL	cCD3^+^, sCD3^−^, CD1a^−^, CD2^+^, CD5^+^, CD7^+^, CD34^−^	NOTCH1/FBXW7 mutations, HOXA, TLX3
Cortical T-ALL	cCD3^+^, sCD3^+/−^, CD1a^+^, CD2^+^, CD5^+^, CD7^+^, CD34^−^	NOTCH1/FBXW7 mutations, TLX1, NKX2.1./2.2, TLX3, TAL/LMO
Early T-cell precursor ALL	cCD3^+^, sCD3^−^, CD1a^−^, CD2^+^, CD7^+^, HLA-DR, CD13, CD33, CD34, CD117	NOTCH1/FBXW7 mutations, HOXA, MEF2C, BCL11B
Mature T-ALL	cCD3^+^, sCD3^+^, CD1a^−^, CD2^+^, CD5^+^, CD7^+^, CD34^−^	NOTCH1/FBXW7 mutations, TAL/LMO

**Table 2:** Antibodies used in ALL therapy.

Therapy	Target	Antibody Type
Blinatumomab	CD19	BiTE
Inotuzumab ozogamicin	CD22	ADC
Rituximab	CD20	mAb
Ofatumumab	CD20	mAb
Epratuzumab	CD22	mAb

BiTE bi-specific T-cell engagers, ADC antibody drug conjugate, mAb monoclonal antibody.

**Table 3:** Combination studies for adult Ph-negative ALL.

Regimen	Indication	N	Median Age	CR/CRi Rate	CR Duration	MRD-Negativity	OS Rate	Reference
Inotuzumab + mini-HCVD ± blinatumomab	R/R ALL	84	35	80%	52% (2-year)	80%	39% (2-year)	[26]
Inotuzumab + CVP	R/R CD22+ ALL	48	43	61%	/	/	10.9 months (median)	[27]
Hyper-CVAD + blinatumomab	Newly diagnosed B-ALL	27	38	100%	RFS 76 %	96%	89% (1-year)	[28]
Hyper-CVAD + rituximab	CD20+, ALL	209	40	92%	/	91%	EFS (2-year) 65%	[29]
Standard/ modified Hyper-CVAD + rituximab	B-ALL	282	41	95%	78%	81%	60% (3-year)	[30]
Hyper-CVAD + rituximab	Newly diagnosed B-ALL	31	46	86%	67% (3-year)	/	89%	[31]
Hyper-CVAD + MTX + cytarabine+ ofatumumab	Newly diagnosed CD20+, B-ALL	69	41	98%	/	65%	68% (4-year)	[32]
Hyper-CVAD + ofatumumab	Newly diagnosed CD20+ B-ALL	222	44	93%	/	93%	66% (4-year)	[33]

HCVD hyper-fractionated cyclophosphamide, vincristine, actinomycin, dexamethasone; R/R relapsed/refractory; ALL acute lymphoblastic leukemia; N number; CR complete remission; CRi complete remission with incomplete hematologic recovery; MRD measurable residual disease; OS overall survival; RFS relapse-free survival; CVP cyclophosphamide, vincristine, prednisone.

**Table 5:** Imatinib/chemotherapy combination studies.

Regimen	Study Population	N	Median Age	CR Rate	HSCT Rate	EFS	RFS	OS Rate	Reference
Imatinib + hyper-CVAD	Newly diagnosed Ph + ALL	54	51	93%	30%	43% (5-year)	43% (5-year)	43% (5-year)	[49]
Imatinib + intensive chemotherapy	Newly diagnosed Ph + ALL (age 15–65)	266	42	92%	72%	33% (4-year)	50% (4-year)	38% (4-year)	[50]
Imatinib + lower-intensity chemotherapy		268	49	98%	62%	37.1% (5-year)	EFS 37% (5-year)	46% (5-year)	[51]

N number; CR complete remission; HSCT hematopoietic stem cell transplantation; EFS event-free survival; RFS relapse-free survival; OS overall survival; Ph+ Philadelphia chromosome positive; ALL acute lymphoblastic leukemia.

**Table 6:** Dasatinib/chemotherapy combination studies.

Regimen	Study Population	N	Median Age	CR Rate	HSCT Rate	EFS	RFS	OS Rate	Reference
Dasatinib + intensive chemotherapy	Ph + ALL	72	55	96%	17%	27 months (median)	44% (5-year)	46% (5-year)	[52]
Dasatinib + lower-intensity chemotherapy	Ph + ALL	71	69	96%	10%	27% (5-year)	EFS 28% (5-year)	36% (5-year)	[53]
Dasatinib + lower-intensity chemotherapy	Ph + ALL	60	42	100%	42%	48% (5-year)	49% (3-year)	58% (3-year)	[54]

N number; CR complete remission; HSCT hematopoietic stem cell transplantation; EFS event-free survival; RFS relapse-free survival; OS overall survival; Ph+ Philadelphia chromosome-positive; ALL acute lymphoblastic leukemia.
